# Evaluation of Direct Colorimetric MTT Assay for Rapid Detection of Rifampicin and Isoniazid Resistance in *Mycobacterium tuberculosis*

**DOI:** 10.1371/journal.pone.0169188

**Published:** 2016-12-28

**Authors:** Gadissa Bedada Hundie, Dawit Woldemeskel, Amare Gessesse

**Affiliations:** 1 Department of Microbial, Cellular and Molecular Biology, College of Natural Sciences, Addis Ababa University, Addis Ababa, Ethiopia; 2 Biotechnology Institute, College of Natural Sciences, Addis Ababa University, Addis Ababa, Ethiopia; Fundació Institut d’Investigació en Ciències de la Salut Germans Trias i Pujol, Universitat Autònoma de Barcelona, SPAIN

## Abstract

With the spread of multidrug-resistant tuberculosis (MDR-TB) strains there is an increasing need for new accurate and cost-effective methods for a rapid diagnostic and drug susceptibility testing (DST), particularly in low-income countries where tuberculosis is hyperendemic. A colorimetric assay using 3-(4, 5-dimethylthiazol-2-yl)-2, 5- diphenyltetrazolium bromide (MTT) has been suggested as a promising method for DST, especially to rifampicin. In this study, we standardized and evaluated the MTT assay for a rapid direct detection of rifampicin and isoniazid resistant *Mycobacterium tuberculosis* strains from sputum specimens using Lowenstein-Jensen (LJ) culture medium as a gold standard. The MTT assay sensitivity, specificity, positive and negative predictive values for rifampicin were 100%, 86%, 100%, 99%, respectively. For isoniazid, the MTT assay had a 100% sensitivity, specificity, positive and negative predictive values. Interestingly, the MTT assay gave interpretable results within two weeks for 94% of the samples compared to 7–14 weeks for LJ media. Overall, an excellent agreement was observed between MTT assay and LJ proportion method (Kappa, 0.91 for rifampicin and 1.00 for isoniazid). In conclusion, the direct colorimetric MTT assay simultaneously detects susceptible and resistant strains of *M*. *tuberculosis* within three weeks. It significantly shortens the time required to obtain a DST result and could be a reliable alternative method for rapid detection of drug-resistant TB strains in high-TB-burden resource-limited settings.

## Introduction

The emergence of *Mycobacterium tuberculosis* (*M*. *tuberculosis*) strains resistant to at least isoniazid and rifampicin, defined as multidrug-resistant tuberculosis (MDR-TB), is a serious cause for concern to TB control programs particularly in low-income countries. The spread of drug-resistant TB significantly worsens the prognosis for achieving a durable cure, presages treatment failure and prolongs the period of transmission potential [1]. Worldwide, the annual MDR-TB burden is estimated at around 500 000 cases or 3.6% of the global TB burden [2]. However, due to lack of laboratory capacity in many developing countries, less than 5% of the existing MDR-TB patients are currently diagnosed [3]. Thus, in many low-income developing countries, the estimated numbers of MDR-TB cases are based on mathematical modeling rather than empirical studies. Ethiopia is among low-income developing countries with high TB burden. The country ranks seventh among the 22 high-TB-burden countries and sixteenth in the list of 27 high-MDR-TB-burden countries [4]. However, Ethiopia has only one laboratory, the National Reference Laboratory, equipped with culture facilities for drug susceptibility testing (DST), and expected to serve a population of more than 100 million. Nevertheless, the *M*. *tuberculosis* culture method is time-consuming due to its slow growth [5]. Hence, rapid, inexpensive and reliable methods for diagnosis and DST in TB are urgently needed.

Several alternative methods are being introduced as potential diagnostic tools in predicting TB infection and DST. The microscopic observation drug susceptibility assay and nitrate reductase assay are relatively rapid methods, yet no suffircient information on their feasibility and costs of implementation [6–8]. Automated commercial mycobacteria detection systems, such as the BACTEC MGIT 960 and BACTEC 460 TB system (Becton Dickinson, Sparks, MD, USA), are promising, but they are expensive, have high running costs, and prone to contamination by other bacteria [9, 10]. DNA based molecular test by polymerase chain reaction (PCR) in sputum samples for diagnosis of TB is currently proposed. However, PCR-based methods are prone to false positivity due to contamination and are very expensive and impractical for routine use in low-income settings like Ethiopia [11–13]. Most recently, GeneXpert MTB/RIF assay (Cepheid, Sunnyvale, CA, USA) was introduced as a rapid and fully automated molecular method to simultaneously detect *M*. *tuberculosis* and rifampicin resistance directly from sputum [14]. This method was endorsed by WHO in 2010, which issued policy recommendations for its application in TB diagnosis in early 2011 [15]. However, due to its high cost, the applicability and feasibility of the GeneXpert MTB/RIF assay in low resource settings remain debatable [16–19]. Thus, for low-income countries, it would be useful to have a simple and inexpensive test that can rapidly detect drug-resistant *M*. *tuberculosis* strains. The colorimetric assay using 3-(4, 5-dimethylthiazol-2-yl)-2, 5- diphenyltetrazolium bromide (MTT) has been developed as a rapid and inexpensive method. It was first introduced by Mossman as a quantitative measure of mammalian cell survival and proliferation [20]. MTT is a yellow tetrazolium salt which can be converted into a purple formazan by dehydrogenase enzyme of living cells. The resulting color can be qualitatively checked visually or measured spectrophotometrically. The assay is based on the principle that the amount of formazan produced is directly proportional to the number of viable cells [20]. The method was later proposed as an indirect method for the detection of rifampicin resistance in *M*. *tuberculosis* [21]. This principle has also been utilized by many other investigators [22–25] for the detection of resistance to other anti-tuberculosis drugs. In addition, the MTT assay has also been proposed as a direct method for rapid detection of rifampicin-resistant *M*. *tuberculosis* from sputum [26]. However, the direct MTT assay was not evaluated under program condition in national TB reference laboratory. Moreover, the assay was not developed for direct detection of isoniazid resistance, one of the most effective first-line drugs used in TB treatment regimens. The aim of this study was, therefore, to examine the MTT assay for detection of isoniazid resistance directly from acid-fast bacilli smear-positive sputum and to evaluate the assay for direct detection of rifampicin and isoniazid resistant *M*. *tuberculosis* using proportion methods on Lowenstein Jensen (LJ) medium as a gold standard.

## Materials and Methods

### Study design and Specimens

This was a cross-sectional study conducted from September 2006 to May 2007 on 154 pulmonary smear-positive TB patients (147 new cases and 7 re-treatment cases) referred to St Peter’s TB Specialized Hospital in Addis Ababa, Ethiopia. A single sputum specimen from each patient was collected in screw capped universal cup and transported in a cold chain to the national TB reference laboratory.

### Chemicals and antibiotics

A stock solution of MTT 5mg/ml (sigma, St. Louis, MO, USA) was prepared in phosphate buffered saline (PBS), pH 6.8, filter sterilized with 0.2 μm filter and kept at 4°C in the dark. A final concentration of 0.5 mg/ml MTT was used in the assay. A formazan solubilization buffer was prepared by mixing 1:1 (vol / vol) 20% sodium dodecyl sulfate (SDS) and 50% dimethylformamide (DMF) (sigma). For the antibiotics, two different stocks of rifampicin (RIF, Sigma) and isoniazid (INH, Sigma) were prepared; one for MTT assay and the other for DST on LJ media. For the MTT assay, a stock solution of 80 μg/ml rifampicin and 1mg/ml isoniazid were prepared in ethanol/PBS and PBS, respectively. The stock solutions were filter sterilized, aliquoted and stored at -20°C until use. The final concentration of 2 mg/ml RIF and 0.2 mg/ml INH were used in the MTT assay. While for the DST on LJ medium, stock solutions of RIF (10 mg/ml) and INH (10 mg/ml) were prepared in DMSO and sterilized distilled water, respectively, and kept at -20°C. RIF and INH were dispensed to 500 ml LJ medium to get a final concentration of 40 μg/ ml RIF and 0.2 μg/ ml INH.

### Mycobacterial culture and isolates identification

All sputum specimens were digested and decontaminated using modified Petroff’s method, which is used routinely in the Ethiopian national TB reference laboratory at Ethiopian Health and Nutrition Research Institute (EHNRI). The pellets were resuspended in 3 ml PBS and an aliquot of 100 μl was inoculated onto two Lowenstein Jensen slants for primary isolation. The tubes were incubated at 37°C and examined weekly for bacterial growth until week eight. From the remaining resuspended sample, an aliquot of 500 μl was inoculated into each of twelve tubes containing 3 ml Middlebrook 7H9 broth supplemented with 10% Oleic acid-Albumin-Dextrose- Catalase (OADC), 0.5% glycerol, and one vial PANTA (a standard antibiotic cocktail that contains Polymyxin B, Amphotericin B, Nalidixic acid, Trimethoprim, and Azlocillin). Briefly, six tubes were used to test RIF resistance and the other six for INH resistance. Three of the six tubes from each group contained either rifampicin (2 mg/ml) or isoniazid (0.2 μg/ml) while the remaining tubes were drug-free controls. All tubes were incubated at 37°C, and MTT assay was done every week for three consecutive weeks. All *M*. *tuberculosis* culture isolates were identified using polymerase chain reaction based on genomic deletion analysis [27].

### Standardization of MTT assay for Isoniazid (INH) resistance

From the stock solution of 100 μg/ml INH, three different final concentrations of INH (0.1, 0.2 and 0.4 μg/ml) were used to determine the INH critical concentration to be used in the direct MTT assay. Ten clinical isolates of *M*. *tuberculosis* with a previously known INH susceptibility pattern by standard proportion LJ media; five INH-resistant isolates and five susceptible were used to standardize the assay. Reference *M*. *tuberculosis* strains, ATCC 27294 (INH-susceptible) and ATTCC 35835 (INH-resistant) were used as experimental controls. Each of the isolates was subcultured onto two LJ media and incubated for 3 to 4 weeks at 37°C. Inocula were prepared by suspension of colonial growth from 3–4 weeks old LJ cultures in sterilized distilled water to a turbidity equal to that of a no. 0.5 McFarland standard. A 0.5 ml of inoculum was inoculated into multiple screw capped tubes containing 3 ml 7H9 broth. Thus, for each isolate tested, panels of 12 tubes with 3 ml of 7H9 broth (3 tubes each for 0.1 μg/ml, 0.2 μg/ml and 0.4 μg/ml INH concentrations, and the remaining 3 tubes drug-free controls) were used. All test tubes were incubated at 37°C until the day of MTT assay.

The indirect MTT assay was carried out as described previously [21]). Briefly, 300 μl of 5 mg/ml MTT solution was added to culture broth and incubated at 37°C for 4 hours. A formazan solubilization buffer (500 μl) was added, and the tubes were mixed on vortex and incubated at 37°C for another hour. Optical density (OD) was measured at 570 nm against a blank containing 7H9 broth, MTT, and solubilization buffer. Relative optical density unit (RODU) values were calculated by dividing the OD of the drug-containing tubes with the OD of drug-free control. To determine the cutoff value for isoniazid, after a thorough vortex, 100 μl of the bacterial suspension was transferred from the 7H9 control medium to the tubes containing LJ medium before doing the MTT assay. The tubes were incubated at 37°C and observed each week for colony forming unit (CFU). The OD value from the MTT result was correlated with CFU to determine the lowest OD value that corresponds to the CFU. A visual reading of color changes from yellow to purple in the control tubes was also compared with CFU. No CFU was observed for broth cultures with < 0.1 OD_570_; therefore, the results were considered interpretable when the OD value of the control was ≥ 0.1.

### Direct MTT assay

The direct MTT assay was done each week for three weeks as described by Abate et al [26]. Briefly, 300 μl of 5 mg/ml MTT solution was added to each test tube containing 3 ml 7H9 broth (i.e., drug-containing tubes, drug-free control tubes and blank control). The tubes were vortexed and incubated for four hours at 37°C. The formazan produced was dissolved in solubilization buffer and the tubes were re-incubated for 1 hour. The change in color from yellow to purple in each tube was recorded visually. The clinical isolates showing color change to purple in both control and drug-containing tubes was labeled as resistant while isolates showing a color change only in drug-free controls but not in drug-containing tubes was labeled as sensitive. Results were confirmed by spectrophotometer (Novaspec II photometer, Pharmacia Biotech Ltd, UK) at 570 nm by measuring the optical density of each tube against a reference blank tube containing 7H9 broth, PBS, MTT and solubilization buffer. The MTT results were defined as interpretable if the OD of control tube was ≥ 0.1. The Relative optical density unit (RODU) was calculated for each sample by dividing the OD of a drug-containing tube by the OD of drug-free control. RODU of < 0.2 was taken as a susceptible result and RODU of > 0.5 was taken as a resistant result. RODU between 0.2 and 0.5 was considered indicative of borderline resistance. Values obtained each week for samples containing susceptible isolates were compared with those of samples containing resistant isolates (using the Mann-Whitney U test). For each sample in 7H9 broth, bacterial contamination was checked by growing subcultures on nutrient agar medium overnight before performing the MTT assay.

### Drug susceptibility testing

For all clinical isolates identified as *M*. *tuberculosis* by polymerase chain reaction (PCR) based on genomic deletion analysis, standard drug susceptibility test was done to rifampicin and isoniazid by the modified proportion method on Lowenstein Jenson media [28]. Drug solutions were added to LJ medium prior to inspissation to achieve final concentrations of 40 μg/ ml RIF and 0.2 μg/ ml INH. Drug-susceptible reference *M*. *tuberculosis* strain, H37Rv, was used as experimental control.

### Statistical analysis

Statistical analysis was performed using SPSS version 13 (Statistical Package for the Social Sciences, Chicago, IL, USA). Mann-Whitney U test was used to detect statistically significant differences between the growth pattern of resistant and susceptible isolates at 95% confidence interval. A probability of < 0.05 was considered significant. Kappa value was calculated to analysis the level of agreement between the standard proportion method and MTT assay.

### Ethical considerations

The study was reviewed and approved by the ethical review committee of Addis Ababa University. Study participants were enrolled after they consented a written informed consent. For the illiterate participants, health professionals informed each participant about the informed consent sheet, and those volunteers and willing to participate by signing the informed consent were included in the study. All the participants consent kept confidential in a secure place. The consent procedures for all participants was also approved by the institutional ethics review committee.

## Results

**Fig 1:**
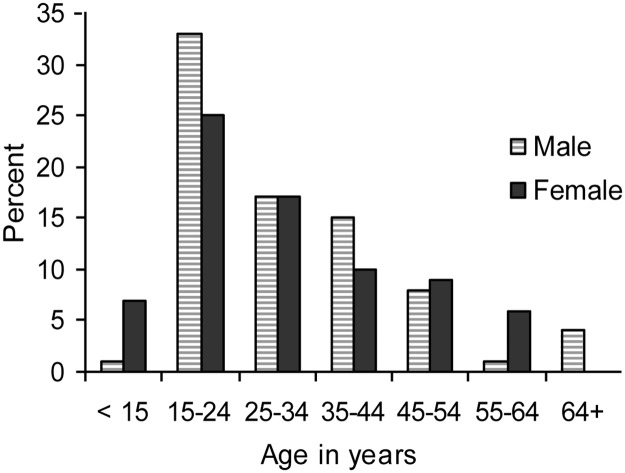
Age and sex distribution of patients.

A total of 154 sputum specimens were collected from 146 new TB patients and 7 re-treatment patients. The mean age of the patients was 30.45 years (range: 7–80 years) and median age 25.5 years (Fig 1). Among the patients, 79 (51.3%) were males and 75 (48.7%) were females.

### *M*. *tuberculosis* culture result by MTT assay and Lowenstein-Jensen media

**Fig 2:**
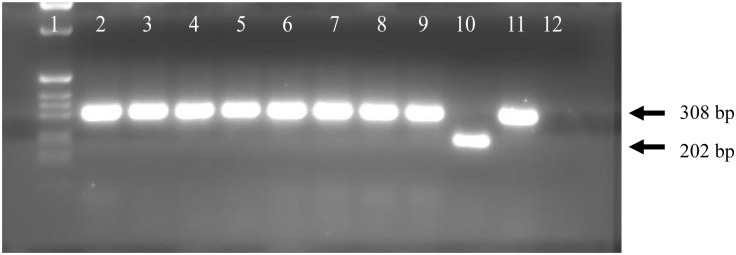
PCR product of clinical isolates. Image of PCR product amplified with RD10 primer; 1 = 1kb ladder, 2–9 = clinical culture isolates, 10 = *M*. *bovis*, 11 = *M*. *tuberculosis*, 12 = negative control (TE).

To evaluate the direct MTT assay, 154 sputum specimens were cultured on LJ media and Middle brook 7H9 broth medium (7H9). On LJ media out of 154 sputum specimens, 133 (86.36%) were culture positive and 14 (9.09%) were culture negative. In the 7H9 broth media used for the MTT assay, 132 (85.71%) were culture positive and 15 (9.74%) were culture negative (OD _570_ < 0.1, in the drug-free control broth). Among the 15 isolates that gave uninterpretable MTT result (OD _570_ < 0.1), 2 had growth on LJ media and were culture positive. From the 14 isolates that were culture negative on LJ media, one grew on 7H9 broth and had MTT result. Based on the polymerase chain reaction genomic deletion analysis, results of all our clinical isolates were confirmed as *M*. *tuberculosis* (Fig 2).

### Standardization of MTT assay for direct detection of isoniazid-resistant *M*. *tuberculosis*

**Fig 3:**
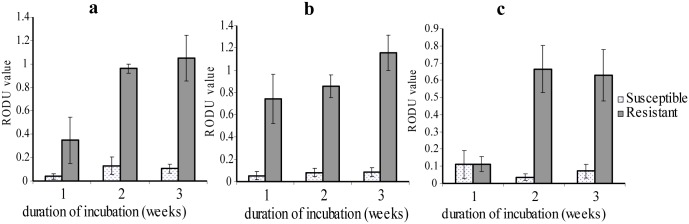
MTT assay growth pattern of *M*. *tuberculosis* isolates at different INH concentration. (a) INH 0.1 μg/ml concentration: growth of isoniazid susceptible isolates RODU mean ± SE (n = 3, first week and n = 5, second and third weeks), and isoniazid resistant isolates (n = 4, first week and n = 5, second and third weeks). (b) INH 0.2 μg/ml concentration: growth of isoniazid susceptible isolates (n = 3, first week and n = 5, second and third weeks), and resistant isolates (n = 4, first week and n = 5, second and third weeks). (c) INH 0.4 μg/ml concentration: growth of isoniazid susceptible isolates (n = 3, first week and n = 5, second and third weeks), and isoniazid resistant isolates (n = 4, first week and n = 5 second and third weeks).

Prior to performing the direct MTT assay for isoniazid resistance detection, the method was standardized using five previously known INH susceptible and five resistant isolates. Three different final concentrations of isoniazid (0.1μg/ml/, 0.2μg/ml, and 0.4μg/ml) were used to determine the critical INH concentration to be used for direct detection of INH resistant *M*. *tuberculosis* in 7H9 broth medium. Growth pattern of *M*. *tuberculosis* at 0.1μg/ml isoniazid concentration indicated that out of the five tested susceptible isolates, three gave interpretable result beginning week one and the remaining isolates at week two and three. Four of the resistant isolates gave interpretable result beginning from the first week and the remaining in the succeeding weeks. The RODU of susceptible isolates remained below 0.2 in all the three weeks of assay except one isolate with RODU above 0.2 (i.e., the susceptible isolate was found borderline resistant at 0.1μg/ml isoniazid concentration) (Fig 3a). The resistant isolate showed RODU < 0.5 in the first week and RODU > 0.5 in the subsequent weeks. The mean ± standard error (SE) RODU value of resistant isolates were 0.82 ± 0.12 and the susceptible isolates were 0.1 ± 0.03. The mean RODU value of resistant and susceptible isolates was not statistically significant at week one (*p* > 0.05) but they showed a significant difference at week two and three (*p* < 0.001).

At 0.2 μg/ml isoniazid final concentration, four resistant and three susceptible isolates gave interpretable result starting from week one and the remaining isolates at week two and three. As shown in Fig 3b, the growth pattern of INH susceptible isolates had RODU < 0.2 and the resistant isolates > 0.5 in all weeks of the assay. The mean RODU value of resistant and susceptible isolates was 0.94 ± 0.11 and 0.08 ± 0.02, respectively. There was a significant difference between the mean RODU of susceptible and resistant isolates at week one, week two and week three of the assay (*p* < 0.05, *p* < 0.001 and *p* < 0.001, respectively).

For the 0.4 μg/ml isoniazid final concentration, three susceptible and four resistant isolates gave interpretable result beginning from week one and the remaining five resistant and five susceptible isolates in the second and third week. The RODU of susceptible isolates remained below 0.2 for all weeks of the assay. The resistant isolates showed mean RODU < 0.5 in the first week and mean RODU > 0.5 in the subsequent weeks assay (Fig 3c). The mean RODU of resistant isolates was 0.51 ± 0.1. Except for one isolate with the RODU value of 0.2 (i.e., the resistant isolate was found susceptible at 0.4 μg/ml isoniazid final concentration) all had RODU above 0.5. The mean RODU value of susceptible isolates was 0.07 ± 0.02. The mean RODU value of resistant and susceptible isolates was not statistically significant for the first week (*p* > 0.05) but was significant for week two and three (*p* < 0.01). The aforementioned results showed that there is one discordant result both at 0.1 μg/ml and 0.4 μg/ml isoniazid final concentrations but not at 0.2 μg/ml. Moreover, at week one the RODU of resistant isolates was almost equal to that of the susceptible isolates (RODU < 0.2), which showed the concentration was too high. Thus, 0.2 μg/ml INH is the final critical concentration to be used in the direct MTT assay.

### Comparison of MTT assay with the standard proportion method on LJ media

Out of the 115 isolates tested for rifampicin resistance, the MTT assay identified 6 (5.2%) as resistant (RODU > 0.5) and 109 (94.8%) as susceptible (RODU < 0.2), whereas the proportion method identified 7 (6.1%) as resistant and 108 (93.9%) as susceptible (Table 1). The drug sensitivity testing results obtained by MTT assay and standard proportion method showed complete agreement for all isolates except for one isolate which was discordant, found susceptible by MTT assay (RODU, 0.09/1.2, = 0.08) but resistant according to the standard proportion method (Table 1). Of the 38 isolates tested for susceptibility to INH, complete agreement between MTT assay and standard proportion method was found for all isolates (32 susceptible and 6 resistant by both methods) (Table 1). Out of 22 isolates tested for both INH and RIF, 4 isolates were identified as MDR-TB, 18 as non-MDR-TB (16 isolates susceptible to both drugs, 2 isolates susceptible to RIF but resistant to INH) by both methods.

**Table 1:** Performance of MTT assay compared with the LJ standard proportion method (PM) in detection of *M*. *tuberculosis* clinical isolates (n = 131^a^).

Drug	Total No. of isolates tested	No. of isolates with indicated result				Efficiency of the MTT assay				
		Susceptible		Resistant						
		MTT	PM	MTT	PM	Specificity(%)	Sensitivity(%)	PPV(%)	NPV(%)	Accuracy^d^
RIF	115	109	108	6	7	100	85.71	100	99.08	0.99
INH	38	32	32	6	6	100	100	100	100	1.00
INH+RIF	22	18	18	4	4	100	100	100	100	1.00

^a^ n = 93 tested only for RIF; n = 16 tested only for INH; n = 22 tested for both drugs

^d^The probability of obtaining a true positive or true negative result

PPV = positive predictive value, NPV = negative predictive value

#### Specificity and Sensitivity of the MTT assay

The efficiency of the MTT assay in comparison with the LJ standard proportion method was shown in Table 1. Specificity, i.e., the ability to detect true drug susceptibility, was 100% both for rifampicin and isoniazid. Sensitivity, i.e., the ability to detect true drug resistance, was 85.71 and 100% for rifampicin and isoniazid, respectively. The overall concordance was 99.3%. There was an excellent agreement between the standard proportion method and MTT assay for drug sensitivity testing of rifampicin and isoniazid (Kappa, 0.91 and 1, respectively).

#### Turnaround time of MTT assay and LJ

The time required to obtain drug susceptibility test results was shorter for the MTT assay compared to the LJ medium (Table 2). Among the 131 samples analyzed, 74 (56.5%) gave interpretable result (OD value of control tube > 0.1) in the 1^st^ week, 123 (93.9%) in the 2^nd^ week, and all 131 (100%) in the 3^rd^ week. Out of the 6 rifampicin resistant isolates detected by MTT assay, 5 (83.3%) gave interpretable result during the second week of incubation and 100% during the third week. All of the 6 isolates found resistant to isoniazid was identified by week two. For both drugs, there is no resistant result at week one. This shows that the results for fully susceptible strains were significantly likely to be available before those strains with drug resistance. Whereas, in the conventional LJ method used it took 7–14 weeks (3–8 weeks for primary culture isolation and additional 4–6 weeks for the DST result).

**Table 2:** Time required by direct MTT assay to detect susceptible or resistant *M*. *tuberculosis* strains to rifampicin and isoniazid.

Weeks of incubation	% of clinical isolates with MTT assay result		NO. of clinical isolates giving interpretable MTT assay result^a^			
	RIF	INH	RIF		INH	
			S	R	S	R
1	53.04	60.5	61	0	23	0
2	93.91	100	102	5	32	6
3	100	100	109	6	32	6
**Total**	**100**	**100**	**109**	**6**	**32**	**6**

^a^ S, susceptible; R, resistant

#### Relative Optical Density Unit

**Fig 4:**
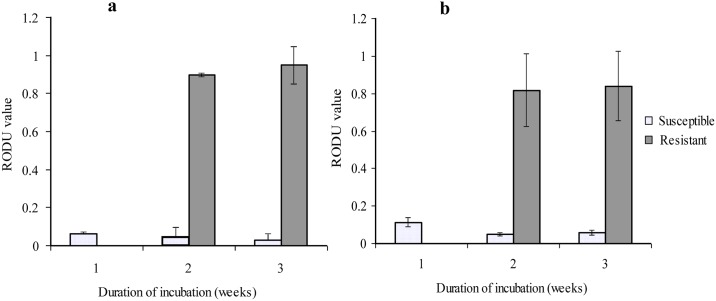
MTT assay growth pattern of *M*. *tuberculosis* isolates in different weeks incubation in drug-containing Middle brook 7H9 broth. (a) Growth patterns of rifampicin susceptible isolates (n = 61 first week, n = 102 second week, and n = 109 third week), and rifampicin resistant isolates (n = 5 second week and n = 6 third week) measured by RODU values (mean ± SE). (b) Growth patterns of isoniazid susceptible isolates (n = 26 first week and n = 38 both in second and third weeks), and isoniazid resistant isolates (n = 6 in the second and the third weeks) measured by RODU values (mean ± SE). The difference in RODU values of resistant and susceptible isolates in each week’s incubation was statistically significant (*p* < 0.0001 {Mann-Whitney U test}).

The RODU, which is the ratio of the drug-containing tube to drug-free control tube, has been used to determine differences in the ability of *M*. *tuberculosis* isolate in reducing MTT and define resistance and susceptibility to rifampicin and isoniazid. Isolates with RODU value above 0.5 are considered as resistant and those with RODU value of below 0.2 as susceptible. In this study, the mean (± standard error, SE) RODU value of resistant isolates was 0.926 ± 0.04 whereas the mean (± SE) RODU value of susceptible isolates was 0.043 ± 0.003 (for rifampicin) (Fig 4a). The mean RODU value of isoniazid-resistant isolates was 0.829 ± 0.11 whereas the mean RODU value of isoniazid susceptible isolates was 0.053 ± 0.009 (Fig 4b). For the resistant isolates, the first week MTT assay was not interpretable (OD value of control tubes < 0.1). The RODU values of samples containing susceptible mycobacteria remained below 0.2 in all weeks of experiments and that the RODU values of samples containing resistant mycobacteria were above 0.5 (Fig 4). The differences in the RODU of samples containing susceptible and resistant isolates were statistically highly significant (*p* < 0.0001 {Mann-Whitney U test}). There was also variation among the resistant isolates in their growth pattern in drug containing 7H9 broth, ranging from an OD value of 0.32 to 1.16.

#### Interpretation of the MTT assay by visual reading

**Fig 5:**
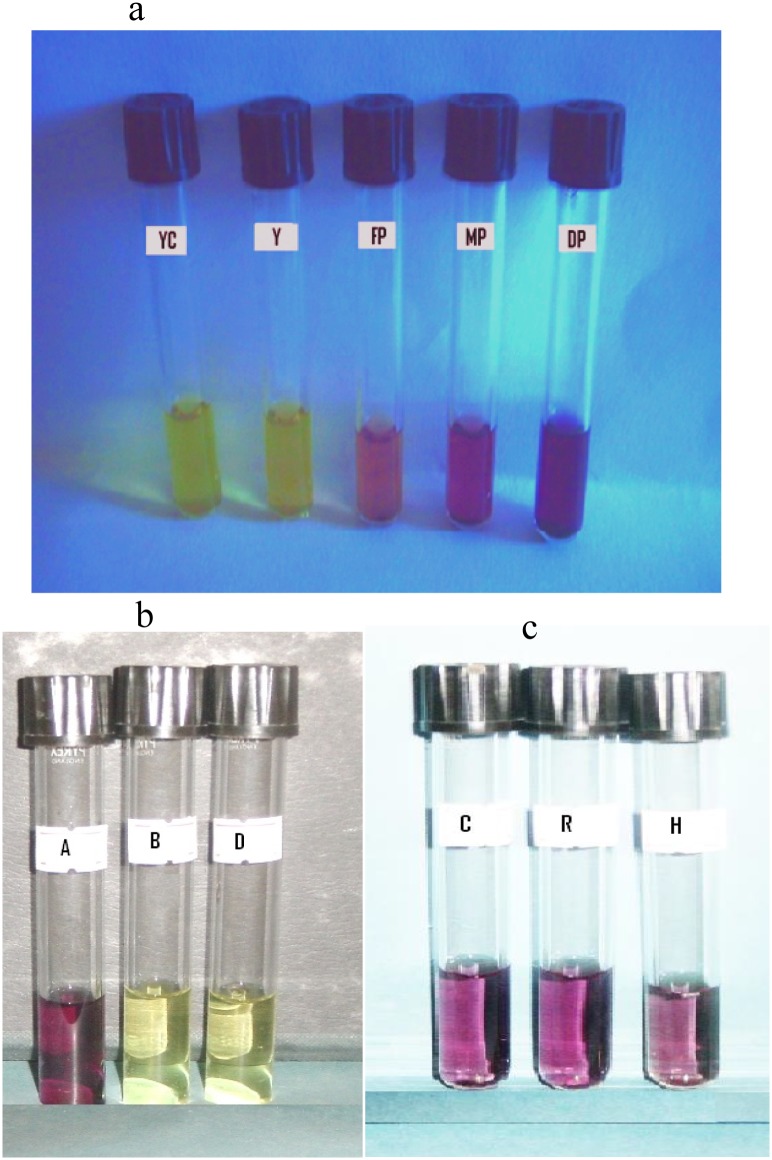
Visual reading of typical color change in the MTT assay. (a) Difference in color change; YC = blank control (without bacteria and drug) with yellow color, Y = Control tube (without drug) with yellow color and corresponding OD value < 0.1, FP = Control tube with faint (light) purple color and corresponding mean OD value 0.23 ± 0.015, MP = Control tube with medium purple and corresponding mean OD value 0.51 ± 0.027, DP = Control tube with dark purple color and corresponding mean OD value 0.99 ± 0.027. (b) Susceptibility visual determination; A = Growth in drug free control tube, B = Rifampicin susceptible, D = Isoniazid susceptible. (c) Resistant visually determination; C = control tube without drug, R = rifampicin resistant, H = isoniazid resistant.

The visual determinations of color change (relative to that of purple in the control tubes) were correlated with RODUs. Cultures regarded as purple by visual observation had OD values of > 0.1 (Fig 5a; FP, MP, and DP). By visual reading of the MTT assay, all drug sensitive TB cultures had a yellow appearance in color (OD < 0.06) (Fig 5b; B and D), and the drug-resistant cultures varied from light to dark purple (OD value ranging from 0.32 to 1.16) (Fig 5c; R and H). RIF-resistant and INH-resistant isolates demonstrated RODU values of, 0.926 ± 0.04, and 0.829 ± 0.11, respectively. This shows that visual reading is sufficient enough to correctly identify resistant and susceptible *M*. *tuberculosis* clinical isolates.

## Discussion

The spread of tuberculosis in general and the emergence of multi-drug resistant (MDR) strains of *M*. *tuberculosis*, in particular, poses a serious challenge to TB control programs. The most widely used conventional method to test drug susceptibility of *M*. *tuberculosis* strain is time-consuming. It requires 3–8 weeks for initial culture isolation and additional 4 to 6 weeks for the actual drug susceptibility test [5]. During this lag waiting period, the patient could suffer and the time for treatment is prolonged. In addition, during this period, the chance of spreading the drug-resistant TB strains to other healthy individuals increases. Hence, there is a need for rapid, affordable, and sensitive predictive screening methods to aid in control of TB and MDR spread particularly in low-income countries like Ethiopia where TB is endemic. The MTT assay described in the present study could prove to be one such diagnostic method.

This study demonstrates that a simple rapid colorimetric MTT assay can be used to determine the susceptibility or resistance of *M*. *tuberculosis* strains to rifampicin and/or isoniazid in a sputum sample directly without the need for primary mycobacteria isolation. This shortens the time required for drug susceptibility reporting by three to nine weeks compared to the standard proportion method. The maximum period required to complete all the investigations with the MTT assay for the 131 specimens analyzed in this study was three weeks. It is also interesting to note that 94% (123/131) of the clinical isolates gave DST result in just two weeks. Other direct rapid methods such as the BACTEC 460 system, the MGIT 960 and molecular methods have a turnaround time less than 12 days [9–13]. However, they are very expensive and impractical for a routine diagnostic use in low-income countries like Ethiopia where more than 80% of the population lives in rural areas. The most recently introduced GeneXpert MTB/RIF represents a major milestone for global TB diagnosis because of its reliability when compared to sputum microscopy and the speed of getting the result when compared with culture method [14]. Nevertheless, the feasibility of this method in low-income setting remain debatable because of its running cost, short shelf life of the cartridges, a need of a very stable electricity supply and annual instrument calibration [16–18].

The MTT assay was evaluated against a gold standard proportion LJ method for drug susceptibility test and was performed under the same conditions and in facilities available for DST that the national TB reference laboratory routinely provides as a service to the community. In this study, an excellent agreement between the MTT assay and the proportion method was found (kappa = 0.91). The MTT assay identified 6/115 (5.2%) isolates as rifampicin resistant and 109/115 (94.8%) as rifampicin susceptible. Results showed 99% concordance with those obtained by the gold standard proportion method of DST. In a similar study, but under different conditions and protocols, Abate et al [26] reported an overall concordance of drug susceptibility result between MTT assay and proportion method as 100%. It is noteworthy to note that in our study more than 95% of the specimens were from new case patients whereas in Abate et al [26] all are from re-treatment cases. In an indirect MTT assay with a mixture of known sensitive, susceptible and unknown clinical isolates, Foongladda et al [22] has also reported a 98.9% correlation between MTT assay and the proportion method. A multicenter study conducted in seven Latin American countries on 30 clinical isolates of *M*. *tuberculosis* with a known drug susceptibility pattern also showed 98% overall agreement between the indirect MTT assay and the proportion method [24].

The MTT assay was previously used in 96-well microtiter plates as indirect detection of isoniazid resistance in *M*. *tuberculosis* [24, 25]. However, this has a disadvantage from the point of view of biosafety because manipulation of plates could generate aerosols. In the present study, we used a screw cap tube format which is not only decreased the hazard but also is the most appropriate for routine use in the simpler laboratory settings. This study was also the first to test isoniazid using direct MTT assay. The MTT assay was adapted for direct detection of isoniazid resistance on sputum specimens and was evaluated against proportion method as a gold standard. The direct MTT assay correctly identified those isolates that were confirmed INH resistant by the proportion method as resistant and the susceptible isolates as susceptible. The sensitivity and specificity of direct MTT assay for INH was fully in agreement with proportion method (100%). This finding shows that the MTT assay could be used directly on sputum specimens without the need for primary isolation on LJ medium unlike the indirect one [21, 29]. Interestingly, this assay reduces the initial 3–8 weeks required for primary isolation and provides result in short period of time.

Unlike the previous study [26], in this study there was no resistant result at week one for both drugs. This shows that the results for fully susceptible strains were significantly likely to be available before those for strains with any resistance. This might be due to the fact that drug-resistant isolates have lower overall metabolic activity and grow slower than the wild strains [30]. Thus, the sensitivity of the MTT method depends on the metabolic activity of viable cells in order to achieve measurable MTT reduction [20].

Another aim of the present study was also to see whether a visual reading of the MTT assay was enough to interpret the MTT assay results in order to make the method simpler to use in resource-limited settings. We observed an excellent agreement between the visual reading and spectrophotometric OD measurements for the growth of *M*. *tuberculosis* in both drug-containing and drug-free medium (Fig 5). Thus, the colorimetric MTT assay result is not particularly vulnerable to observer misinterpretation, and visual reading of results could be sufficient enough where spectrophotometer is not available.

In conclusion, the direct colorimetric MTT assay is a simple, rapid and inexpensive diagnostic and susceptibility test method for *M*. *tuberculosis* that can be completed within three weeks. Visual reading or spectrophotometric reading can be used to determine the growth of bacteria in the medium. Due to its high levels of agreement with the standard golden culture method, the MTT assay has the potential to provide rapid detection of RIF and INH-resistant *M*. *tuberculosis* in routine clinical use in low-income countries.
