# Correction: Evaluation of Direct Colorimetric MTT Assay for Rapid Detection of Rifampicin and Isoniazid Resistance in *Mycobacterium tuberculosis*

**DOI:** 10.1371/journal.pone.0171964

**Published:** 2017-02-06

**Authors:** Gadissa Bedada Hundie, Dawit Woldemeskel, Amare Gessesse

There is an error in the penultimate sentence of the Materials and Methods section under the subheading “Chemicals and antibiotics.” The correct sentence is: The final concentration of 2 μg/ml RIF and 0.2 μg /ml INH were used in the MTT assay.

There is an error in the antepenultimate sentence of the Materials and Methods section under the subheading “Mycobacterial culture and isolates identification.” The correct sentence is: Three of the six tubes from each group contained either rifampicin (2 μg/ml) or isoniazid (0.2 μg/ml) while the remaining tubes were drug-free controls.
